# Medication Error- A Case Report of Misadventure with Methotrexate

**Published:** 2018-06-30

**Authors:** Anup Singh, Aru Chhabra Handa

**Affiliations:** 1 Department of Otorhinolaryngology and Head & Neck Surgery, Medanta- The Medicity, Gurugram, Haryana, India

**Keywords:** *drug overdose*, *Methotrexate*, *mucositis*, *pancytopenia*

## Abstract

Methotrexate is an antimetabolite drug with antineoplastic and immunomodulatory properties, useful as an antineoplastic agent in various haematological and solid tumours. MTX toxicity can occur because of accidental ingestion/overdose by the patient or because of prescription error. The toxic effects manifest as severe mucositis or as organ damage (bone marrow depression, renal/ hepatic injury). The toxicity usually results from parenteral overdose or repeated chronic drug ingestion. Acute high dose ingestion does not result in MTX toxicity because of saturable absorption kinetics. We present a case of MTX toxicity occurring as a result of prescription error resulting in repeat daily dosing of the drug, and the challenges associated with the management of the same, in a patient with multiple comorbidities. The present case emphasizes on a note of caution on the part of the prescriber and the suggestions regarding the measures which can be taken to avoid MTX toxicity.

## INTRODUCTION

Methotrexate (MTX) is an antineoplastic and immunomodulatory agent. It competes with folic acid for absorption from the gastrointestinal tract and intracellular metabolism involving the conversion of Dihydrofolate (FH_2_) to Tetrahydrofolate (FH_4_) and diminishes the availability of the latter as a reducing agent for DNA and RNA synthesis.^1^

The major manifestations of MTX toxicity are severe mucositis, bone marrow depression, renal and liver dysfunction. The toxicities are usually as a result of parenteral administration or repeated oral ingestion.^2^ Single oral dose usually does not result in significant toxicity in a patient with normal renal functions.^3,4,5^

We present a case of MTX toxicity occurring as a result of prescription error, and the challenges associated with its management, in a patient with multiple comorbidities. The present case emphasizes on a note of caution on the part of the prescriber and the suggestions regarding the measures which can be taken to avoid MTX toxicity.

## CASE REPORT

**Figure 1.**
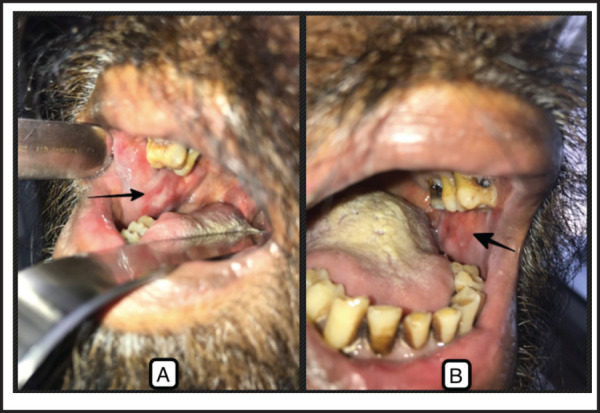
Superficial, erythematous, well defined, non-indurated, mucosal ulcerative lesions involving bilateral buccal mucosa.

A 57 years old diabetic male was referred to us, by the Department of Respiratory Medicine at our institute, with nasal pain for 4–5 days. The patient had a history of pneumonia with sepsis from which he had recently recovered. He had multiple comorbidities including Type 2 Diabetes Mellitus for 25 years, Coronary Artery Disease for three years with a history of percutaneous transluminal coronary angioplasty and End Stage Renal Disease for three years. The patient was on injectable insulin for diabetes management and on alternate day maintenance haemodialysis for last 3 months, awaiting renal transplantation. On nasal endoscopy, the patient was found to have blackish discoloration over anterior end of middle turbinate for which a nasal endoscopie biopsy was done under local anaesthesia. The histopathological report came out to be broad aseptate hyphae suggestive of mucurmycosis. Under general anaesthesia, excision of the middle turbinate and limited functional endoscopic sinus surgery was done till the bleeding margins were achieved and the patient was started on injection liposomal Amphotericin B in a daily dose of 200 mg to a cumulative dose of 4 grams. After ten days of hospitalization, the patient was discharged in stable condition. Two weeks later, the patient was admitted with multiple oral ulcerations and severe odynophagia. There was no history of fever, bowel or urinary symptoms. The ulcers were superficial and involving multiple sites in the oral cavity including lips, bilateral buccal mucosa and soft palate with a well-demarcated border (Fig 1).

**Figure 2.**
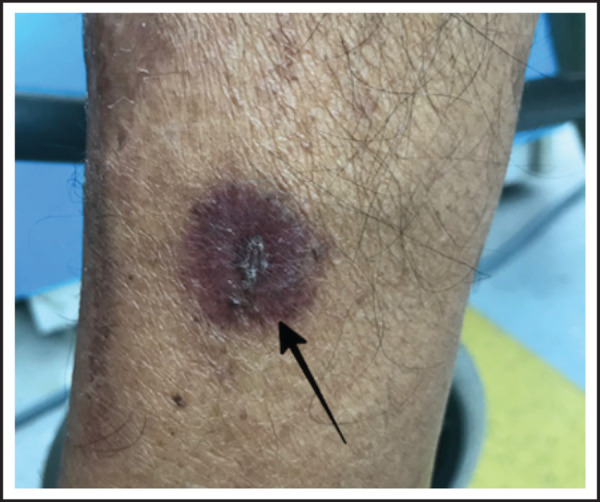
Well defined, erythematous to dusky red, slightly raised circular plaque of 1.5 cm diameter, with central black and white necrotic scale crust on medial aspect of lower leg.

There was a well-defined erythematous to dusky, slightly raised, circular plaque of 1.5cm diameter with central black and white necrotic scale crusts on medial aspect of lower leg (Fig 2).

A review of the drugs being taken by the patient showed that the patient was taking tablet Folitrex (Methotrexate) 5 mg daily for last four days. On further probing, it was found out that patient was previously taking tablet Folic acid and was mistakenly started taking Folitrex in place of Folic acid few days after discharge when folic acid tablets available with the patient had been consumed. The patient was admitted to inpatient care and Ryle's tube was inserted for feeding.

Routine blood tests showed deranged liver and renal function tests (SGOT-174 U/L, SGPT- 200 U/L, Total proteins- 5.7 gm/dl, Serum Albumin- 2.63 gm/dl, Urea-198 mg/dl, Serum Creatinine- 5.3 mg/dl, Na + - 130 mmol/L, K+- 4.5 mmol/L) and alternate day haemodialysis was continued with added human albumin (20%) in hemodialysate. Serial complete blood count showed pancytopenia and the blood picture deteriorated to WBC count- 860//vl, Hb- 7.9gm/dl and Platelets- 30,000//vl on day-3 of admission. In view of low WBC counts, the patient was started on prophylactic intravenous injectable antibiotics (Meropenem and Teicoplanin) and intravenous liposomal Amphotericin-B 50 mg on alternate days. Granulocyte colony stimulating factor (G-CSF) infusion was started in a daily dose of 300 mg subcutaneously and Injection Leucovorin (folinic acid) in a dose of 15 mg IV every six hours. On day 6 of admission, the blood counts started rising and by day-9, the counts had normalized. Injection Leucovorin and G-CSF were stopped after day-4 of starting them.

On day 6 of admission, the patient developed cough with blood tinged sputum and a chest X-ray showed Pleural effusion with concomitant chest consolidation. A diagnostic tapping of the pleural fluid was done and sent for Cell count, glucose/ protein levels, Lactate Dehydrogenase, Adenosine deaminase and Gene Xpert. The tests were suggestive of exudative effusion (probably due to hypoalbuminemia or chronic renal failure). In view of the recurrent collection, an intercostal drainage tube was put on the right side. Oral feeding was resumed from day 7 and Ryle's tube was taken out next day. On the request of the patient and the family, he was discharged on day 13 of admission. At the time of discharge, the blood counts were normal and the patient was feeding orally. The chest tube was subsequently taken out after three days in outpatient setting.

## DISCUSSION

Methotrexate (MTX) is an antineoplastic drug belonging to antimetabolites class (of antifolate type) which, by inhibiting cell-mediated immune reactions, exhibits immunomodulatory properties also. Its principal mechanism of action is via competitive inhibition of the intracellular enzyme ‘dihydrofolate reductase’ (DHFR) which decreases the availability of tetrahydrofolate (FH_4_), needed for synthesis of thymidylate and purines resulting in cell cycle arrest in S-phase.^1^ The differential action of MTX on rapidly dividing tumour cells, epithelial cells, and synovial macrophages is because of enhanced polyglutamation capability of these rapidly dividing cells. The polyglutamated form of MTX gets entrapped inside the cell and has a higher affinity than dihydrofolate and non/monoglutamated MTX for the enzyme DHFR. MTX is bound 30–50% to the plasma proteins and is principally excreted by renal route (|90%) unchanged via both glomerular filtration and tubular secretion. Remaining 10% is excreted in faeces after being metabolized in the liver.^6^

MTX Toxicity manifests as follows:

1. Gastrointestinal toxicity - resulting in stomatitis, mucositis (superficial ulcers varying in number and size on an erythematous background), nausea, vomiting and reduced oral intake.
2. Myelosuppression - resulting in pancytopenia with the increased risk of systemic infections/ sepsis and bleeding.
3. Liver toxicity - resulting hepatic parenchymal injury with the rise of transaminases in acute cases and hepatic fibrosis in chronic cases.
4. Renal toxicity - resulting in acute renal failure, which further decreases the clearance of MTX from the system.
5. Pulmonary toxicity leading to pneumonia and respiratory failure may occur in acute cases, while pulmonary fibrosis may result as a side effect of chronic use.

Predisposing factors for MTX toxicity include deranged renal functions, hypoalbuminemia, elderly patient and co-administration of other medicines which can either displace MTX from plasma bound proteins and prevent its excretion via tubular secretion (Salicylates and NSAIDs, penicillins) or with same enzymatic inhibition mechanism as MTX (Trimethoprim-sulfamethoxazole).^7^ Our patient was an elderly male with end-stage renal disease awaiting renal transplantation, which predisposed him to manifest the drug toxicity.

Common causes of MTX toxicity are either because of accidental ingestion/ overdose by the patient or electronic prescription error by physician/ pharmacist.^8^ The drug exposure may occur as either chronic daily low dose ingestion or as accidental/ deliberate acute ingestion. The usual dose of MTX as a weekly tablet makes it prone to overdose with daily medication because of prescription, dispensing or administration errors. Misinterpretation of Folic acid with Folitrex (MTX) in our case led to the patient receiving a daily dose of MTX which resulted in patient manifesting toxicity in the form of stomatitis (gastrointestinal toxicity), pancytopenia (Myelosuppression) and transaminitis (deranged liver functions).

It is important to note that acute high dose ingestion of MTX rarely leads to clinically significant toxicity, since oral administration of MTX exhibits a saturable absorption kinetics.^9^ This, along with a rapid distribution half-life (2 hours) and elimination half-life (6–8 hours) results in an almost undetectable level of MTX in plasma after 24 hours of single dose ingestion. Instead, a chronic daily ingestion of weekly dose results in clinically significant toxicity.

MTX clearance is proportional to the renal function and with impaired renal function, even a single low oral dose of MTX may result in significant systemic adverse effects.^10^ On the other hand, prolonged duration of the drug in plasma may result in precipitation of MTX crystals in renal tubules and result in renal dysfunction which, in a vicious cycle, will further beget MTX toxicity. The renal damage, hence, can be a cause or consequence of MTX toxicity.^11^

The strategies for managing MTX toxicity are:

1. Drug elimination from body:
  - Adequate hydration and urinary alkalinization: for rapid elimination of drug via renal route. The acidic pH (<5.5) results in precipitation of MTX and its 7-hydroxy metabolite. However, an increase of pH to 7 increases the solubility of MTX 10 times and helps in effective renal elimination of the drug.^12^
  - Glucarpidase: is a carboxypeptidase, approved by U.S. food and drug administration for cases of MTX toxicity with plasma levels >1 /vmol/L in cases with renal failure. This drug metabolizes plasma circulating MTX to an inactive form and reduces plasma levels of the drug by around 97% within 15 minutes. However, it fails to metabolize the intracellular MTX and hence concomitant folinic acid needs to be administered.^7^
2. Leucovorin (folinic acid) rescue: given orally in 24 hours and parenterally within 30–36 hours of overdose, results in repletion of reduced intracellular folate pool by bypassing the enzyme DHFR.^13^ For staggered ingestion over >36 hours and in cases of acute ingestion with renal failure, folinic acid is recommended in a dose of 15 mg orally followed by 15 mg intravenously every six hours for three days.^9^
3. Organ-specific damage repair: Management of myelosuppression requires broad spectrum antibiotics and antifungals to be started empirically for febrile neutropenia. Packed RBCs and platelet transfusion may be needed for severe anemia and thrombocytopenia. Recombinant Granulocyte-colony stimulating factor may be given subcutaneously to overcome severe neutropenia by stimulating bone marrow production and maturation of neutrophils.^14^

A bioavailability of <1 gm/m^2^ or the plasma concentration of <10 /vmol/L at 24 hours or <0.1 /vmol/L at 48 hours does not warrant the folinic acid rescue.^15^ The high dose injections (> 1 gm/m^2^), are usually given intravenously in various cancer chemotherapy regime and these are the cases which require nomogram based folinic acid rescue depending on serum levels of MTX. Acute ingestion (accidental/ deliberate) of a single dose in a patient with normal renal functions does not need any specific treatment because the bioavailability of MTX in such circumstances does not exceed 15mg/m^2^ as a result of its saturable pharmacokinetics. However, a patient with impaired renal functions (eGFR<45ml/ min/1.73m^2^) or staggered ingestion over >36 hours shall potentially need specific treatment for MTX toxicity since the drug in such cases stays for a longer duration in the system.^9^ It appears that the duration of exposure of the drug in the system rather than peak plasma concentration is a far more important determinant of systemic MTX toxicity. The low bioavailability, rapid cellular uptake, short distribution half-life and rapid renal excretion make the measurement of serum MTX level measurement unusable for either acute or chronic ingestion. In such cases, measurement of serum levels of MTX does not provide clinically important information from the management point of view. In cases of chronic ingestion, even very low serum levels can be associated with fatal toxicities.^10^

To reduce the incidence of harm associated with MTX errors certain steps are advisable.^16^ The packaging may be labelled with a strict alert regarding weekly dosing of the drug, stating clearly that daily ingestion may result in toxicity. The drug packaging itself may be adjusted such that one pack of MTX for monthly supply contains only weekly doses of the medicines (i.e. 4 doses). Another potential modification may be with a peculiar colour coding of MTX tablet since currently, both MTX and folic acid tablets are dispensed as a small yellow tablet. A strict vigilance on the part of prescriber and dispenser is needed to lower the incidence of such mishappening.

**Consent: JNMA Case Report Form was signed by patient's father and the original is attached with the patient chart.**
